# Economic burden of osteoporotic fractures in Austria

**DOI:** 10.1186/2191-1991-2-12

**Published:** 2012-06-27

**Authors:** Hans Peter Dimai, Kurt Redlich, Monika Peretz, Fredrik Borgström, Uwe Siebert, Jörg Mahlich

**Affiliations:** 1 Department of Internal Medicine, Division of Endocrinology and Metabolism, Medical University of Graz, Graz, Austria; 2 Department of Internal Medicine III, Division of Rheumatology, Medical University of Vienna, Vienna, Austria; 3 Association for the Promotion of Continuing Medical Education and Research, Vienna, Austria; 4 I3 Innovus, Stockholm, Sweden; 5 Medical Management Centre, Karolinska Institutet, Stockholm, Sweden; 6 Department for Public Health and Health Technology Assessment, UMIT–University for Health Sciences. Medical Informatics and Technology, Hall i.T, Austria; 7 Department of Economics, Faculty of Business, Economics and Statistics, University of Vienna, Vienna, Austria

**Keywords:** Burden of illness, Costs of illness, Osteoporosis, Austria

## Abstract

**Objective:**

Osteoporotic fractures impose a huge economic burden on society. Though several cost of illness studies from other countries exist, no equivalent study has been conducted in Austria. Our study aims at assessing costs resulting from osteoporotic fractures in Austria in the year 2008 from a societal perspective.

**Methods:**

We took both direct and indirect costs into consideration. Direct costs encompass medical costs such as expenses for pharmaceuticals, inpatient and outpatient medical care costs, as well as other medical services (e.g., occupational therapies). Non-medical direct costs include transportation costs and medical devices (e.g., wheel chairs or crutches). Indirect costs refer to costs of productivity losses due to absence of work. Moreover, we included costs for early retirement and opportunity costs of informal care provided by family members. For our analysis, we combined data of official statistics, expert estimates as well as unique patient surveys that are currently conducted in the course of an international osteoporotic fracture study in Austria.

**Results:**

For the year 2008, the total annual financial burden incurred by osteoporotic fractures in Austria amounted to approx. €685.2 million, the largest fraction of which was due to the opportunity cost of family care (30.2%), followed by costs for hospitalization (26.6%).

**Conclusions:**

The financial burden of osteoporotic fractures in Austria is substantial. Our findings may have implications for future economic analyses, and also support health care authorities in their decision making.

## Background

Osteoporosis is defined as a „systemic skeletal disease characterized by low bone mass and microarchitectural deterioration of bone tissue with a consequent increase in bone fragility and susceptibility to fracture” [1]. The average lifetime risk in a 50 year old person to experience an osteoporotic fracture has been estimated at 40-50% for women and at 13-22% for men [2]. Accordingly, it has been estimated that in the year 2000 some 9 million osteoporotic fractures have occurred worldwide, including 1.6 million hip fractures, 1.7 million forearm fractures, and 1.4 million clinical (symptomatic) vertebral fractures [3]. These major osteoporotic fractures have been shown to be associated with significant morbidity, and hip and vertebral fractures have been shown to be associated even with excess mortality [4]. Furthermore, the combined annual costs of all osteoporotic fractures have been estimated to be $20 billion in the United States and €30 billion in the European Union, indicating that in addition to morbidity and mortality, osteoporotic fractures are also associated with a significant financial burden to the society [5].

Assessment of the national financial burden of osteoporosis is usually undertaken by means of cost-of-illness studies [6], and several of such have been conducted throughout a number of countries worldwide, particularly in countries of the so called Western World [7-12]. However, the majority of these studies have primarily focused on costs occasioned by hip fractures, since national data on the epidemiology of this type of fracture are more likely to be readily accessible than data from any other type of osteoporotic fracture [3,4,6]. Furthermore, due to differences in healthcare systems, approaches utilized to estimate the number of fractures nationwide, methods of pricing and reimbursement, types and qualities of data as well as computations, results of cost-of-illness studies among different countries can hardly be compared [6]. However, irrespective of such uncertainties, it has been widely accepted that osteoporotic fractures impose a huge economic burden worldwide, with a trend towards further increases [6].

Austria counted some 8.3 million inhabitants at the beginning of 2008. Similar to many other European countries, Austria’s age pyramid at the beginning of the twenty-first century shows a narrow base due to a reduction in birth-rates, whereas the percentage of the senior population 50 years of age and older, is increasing. Within the elderly Austrian population, the number of women clearly exceeds the number of men which is due not only to the higher life expectancy of women, but also to the large number of men who died in World War II.

The incidence of osteoporotic fractures in the Austrian population has been estimated to be among the highest worldwide [13]. For example, in 2008 the total number of hip fractures in the population 50 years and above was 15,615, with an age-standardized incidence of hip fractures for the female population of 605 per 100.000, and an age-standardized incidence of hip fractures in the according male population of 261 per 100.000 [13]. Consequently, it has been hypothesized that the financial burden of osteoporotic fractures in Austria must be substantial.

The aim of the present study was to estimate the financial burden of osteoporotic fractures in the Austrian population, including fractures of the hip, vertebrae, distal forearm, humerus, and ribs.

## Methods

### Direct and indirect costs

To estimate the financial burden, both direct and indirect costs related to osteoporotic fractures have been considered, except costs for rehabilitation. Direct costs in general encompass medical as well as non-medical costs directly related to the causative disease. Accordingly, we included costs for pharmacotherapy, hospital and nursing home, as well as costs for (follow-up) radiographs, wheel chairs, crutches, transport to outpatient settings etc. In contrast, indirect costs, sometimes also referred to as productivity costs, are a measure of present and/or future productivity losses caused by a disease. Indirect costs were calculated according to the human capital approach [14]. Hence, we included productivity loss due to absence from work as well as costs for early retirement and costs of informal (unpaid) care provided by family members and/or other persons. We decided to draw on the human capital approach rather than on the friction-cost method because the former is grounded in neoclassical economic theory while the latter is not [15]. Moreover, Austria’s unemployment rate in 2008 was among the lowest in Europe. This fact served as an additional argument to not employ the friction cost approach in our study. Only in case of labour market imperfections and high unemployment the friction cost approach might be considered as a feasible alternative.

### Source and quantity of costs following fracture

Source and quantity of costs following the first year after fracture have been obtained from the Austrian branch of the International Costs and Utilities Related to Osteoporotic Fractures Study (ICUROS). The ICUROS is an ongoing international study supported by the International Osteoporosis Foundation (IOF) which aims at assessing the consequences of osteoporotic fractures in terms of costs and health related quality of life (QOL) in a standardized form, making results comparable among the different participating countries worldwide [data unpublished, http://www.medscinet.com/icuros/project.aspx]. To assess sources and quantities of costs for the prevailing study, an interim analysis of the Austrian study arm of the ICUROS was performed. Data were extracted from a total of 916 patients who had finished phase II of the study, i.e. who had at least one follow-up interview within the first year after the fracture. Of these patients, 488 had a hip fracture, 158 had a clinical vertebral fracture, 140 had distal forearm fracture, and 122 had a fracture of the humerus. Information on following parameters was obtained: Number of outpatient visits at the primary care level and/or hospital, number of home visits, number of phone counselling, use and dosage of drugs, number and type of community services and other services (e.g. nursing), information on working situation, type of employment (also part or full-time), number of sick days, informal care, information on investments directly or indirectly caused by fracture and its consequences (wheelchair, walking aids, modifications to house/apartment, special utensils for personal hygiene etc.).

Depending on the source of costs, costs themselves where then obtained from institutions and services which most likely would have readily accessible the according information.

### Number of patients with osteoporotic fractures

The number of patients with osteoporotic fractures was calculated for the entire Austrian population. The following types of fracture were included: hip fracture, humeral fracture, clinical (symptomatic) vertebral fracture, distal forearm fracture, and rib fracture. Since most but not all of the fractures may have been due to osteoporosis per se, specific weighting factors have been applied [11].

#### Hip fractures

The number of patients with hip fractures in Austria in 2008 was obtained from the Austrian Hospital Discharge Register (AHDR) for the entire population. As one hundred percent of people who sustain a hip fracture are admitted to hospitals, the number obtained from the AHDR is equivalent to the total number of patients who sustained a hip fracture in 2008. To correct for possible multiple registrations (within a year after admission) for the same diagnosis, a correction factor (0.9) has been applied. These data have been published recently [13].

#### Humeral fractures

The number of patients with humeral fractures admitted to hospitals in Austria in 2008 has been drawn from the AHDR. In contrast to patients who sustain a fracture of the hip, only a part of those who sustain a humeral fracture are admitted to hospitals. The proportion of patients who have been treated in an outpatient setting was assessed by extracting databases from seven large Trauma Units in Austria who covered about 20% of all humeral fractures that occurred in 2008 nationwide. Based on the data obtained from these Trauma Units, it has been estimated that the proportion of humeral fracture patients who have been admitted to hospitals (and hence captured by the AHDR) is 57%. An according correction factor was applied.

#### Clinical vertebral fractures

The number of patients with clinical vertebral fractures who have been admitted to hospitals in Austria in 2008 was derived from the AHDR. However, since not all patients with clinical vertebral fractures are admitted to hospitals, this number would reflect only part of the total number of patients. According to data derived from a recently published Swedish study, it was estimated that the number of patients with clinical vertebral fractures admitted to hospitals account for approximately 10% of all female patients with clinical vertebral fractures, and 15% of all male patients nationwide [16]. To estimate the total number of Austrian patients who sustained a clinical vertebral fracture, an according correction factor was applied.

#### Forearm fractures

The number of patients with forearm fractures who have been admitted to hospitals in Austria in 2008 was derived from the AHDR. Again, this number would reflect only part of the total number of patients who had sustained a forearm fracture, since many of these patients may be treated in an outpatient setting. Several studies have indicated that in general only approximately 20% of patients with distal forearm fractures are admitted to hospitals and treated in an inpatient setting, e.g. for the purpose of operative intervention [17-19]. To estimate the total number of Austrian patients who sustained a forearm fracture, the according correction factor was applied.

#### Rib fractures

The number of patients with rib fractures who have been admitted to hospitals in 2008 in Austria was derived from the AHDR. Again, this number would reflect only part of the total number of patients who had sustained a rib fracture that came to clinical attention [20]. In a Finnish population it has been estimated that only about 10% of all rib fractures after minimal trauma would be admitted to hospitals [21]. To estimate the total number of Austrian patients with low trauma rib fractures, the according correction factor was applied.

### Costs of hospitalization

Data on the average duration of stay in hospital per fracture type (i.e. hip, clinical vertebral, humerus, forearm, ribs) was obtained from the ICUROS, and/or provided by Statistics Austria. To estimate the total number of days of stay in hospital, the number of days of stay in hospitals per fracture type was multiplied by the number of patients who had sustained an according fracture under consideration of correction factors where applicable (see above), and results obtained from the different fracture types were then summarized.

Data on costs per day of stay in hospital were provided by the Austrian Federal Ministry of Health. In average, in 2008 the cost of one day spent in an orthopaedic or trauma department of an Austrian Hospital was € 592 (including medications, nutrition etc.). This value was then multiplied by the total calculated hospital days of stay which have been caused by osteoporotic fractures.

### Costs of outpatient treatment

Costs incurred through consultations of practicing and/or hospital employed specialists (e.g. orthopaedists) were calculated using data provided by the Central Association of Austrian Social Security Institutions**.** In average, costs for consultation of an orthopaedist in a hospital outpatient clinic were € 53.55 per consultation, and costs for consultation of an orthopaedist in the primary care setting were € 73.10 per consultation. These costs were then multiplied by the according number of consultations.

### Other medical and non-medical costs

Number of radiographs, physical therapies and transportation as consequences of a fracture were obtained from the ICUROS database. Unit costs for radiographs, bone mineral density measurement (Dual x-ray Absorptiometry [DXA]) and physical therapy were provided by the Central Association of Austrian Social Security Institutions. Average unit cost of BMD-measurement (lumbar spine and hip) was estimated at € 35.00. Average unit cost of a radiograph was estimated at € 80.40, and the average unit cost of a standard physical therapy was estimated at € 30.00. Information on costs of non-emergency transportation was provided by a common Austrian Ambulance Service (Samariterbund). Mean cost per transportation was estimated at € 57.00. Unit cost for wheelchairs, wheeled walkers, crutches, ramps, cranes etc. were obtained through information provided by the according retail stores.

### Costs of pharmacotherapy

Costs of pharmacotherapy were calculated based on the estimated number of incident osteoporotic fractures in Austria in 2008. However, since data from the Austrian study arm of the ICUROS indicates that only about 22% of patients with recent osteoporotic fracture would receive adequate osteoporosis treatment (data unpublished), estimation of costs of pharmacotherapy only took into account this proportion of patients. Furthermore, to further improve accuracy of estimation, we also considered the proportional distribution of osteoporosis drugs as derived from the ICUROS database. For estimation of costs incurred by analgesics, calcium and vitamin D, again data from the ICUROS were extracted which indicated that ~37% of patients following an osteoporotic fracture would receive analgesic therapy, and 40% would receive calcium and vitamin D. Drug prices were based on the 2008 version of the Austrian EKO (Erstattungskodex) [22].

### Costs of nursing and informal care

Number of hours of nursing care and informal care as a consequence of osteoporotic fracture was obtained from the Austrian ICUROS database. Information on costs of nursing was provided by Statistics Austria, and the average cost of one hour of nursing was estimated at € 30. Information on number of hours of informal (unpaid) care provided by family members or other persons as a consequence of osteoporotic fracture was obtained from the Austrian ICUROS database. Cost of one hour of informal care was estimated at € 27.50, reflecting the average hourly wage of an Austrian employee [23].

### Costs of absenteeism

Data regarding number and costs of sick-days due to osteoporotic fractures in the Austrian population were provided by Statistics Austria and the Austrian Pension Insurance Authority. In total, 3.490 men and 2.336 women had been on sick-leave due to such conditions in 2008. The average number of sick-days in these patients was 16.3 in men, and 18.9 in women. To estimate the costs of absenteeism, the mean annual gross salary per capita of Austrian employees was divided by 220 working days, and this number was then multiplied by the average number of osteoporotic-fracture associated sick-days. However, it should be considered that the costs estimated as described above, would rather underestimate the true costs, as wages alone would not include for example loss of profit or the employer’s contribution to the Austrian Social Security institutions. Therefore, to estimate the total loss of added value incurred through absenteeism, data from the official Input–output Table (IOT) were extracted. Data were provided by Statistics Austria. By means of this analysis, information on so called second round effects was obtained. Second round effects typically occur due to reduced productivity in one company or institution (e.g. caused by absenteeism of employees), with the consequence of decreasing output in supplying companies or institutions [24].

### Costs of early retirement

According to data provided by the Federal Ministry of Labour, Social Affairs and Consumer Protection, as well as Statistics Austria, a total of 338.462 Austrians were registered as in early retirement due to disability (also referred to as disability pension) in 2008 [25], and 25.146 cases of early retirement have been newly registered throughout the year 2008. The average annual cost of early retirement per capita in 2008 was €13.395. Musculoskeletal and connective tissue diseases have been the underlying cause in 6.989 cases, of whom 0.338% was due to osteoporosis-related conditions [25]. Accordingly, the total number of disability pension cases due to osteoporosis-related conditions in 2008, was estimated at approximately 1.444. To calculate the total costs of early retirement due to osteoporosis-related conditions, the estimated number of cases was multiplied with the average annual costs per capita to come up with an estimated amount of € 19.3 million.

## Results

The total cost of osteoporotic fractures in Austria from a societal perspective is € 685.6 million (Table 1). The two biggest cost drivers are the opportunity costs of family care and the cost of hospitalization. The cost of absenteeism in early retirement is rather low which reflects the fact that most patients suffering from an osteoporotic fracture have already left the labour market before the fracture has occurred.

**Table 1:** Direct, indirect, and total costs

		**Costs (in € Mio)**	**Propotion**
Direct costs	Inpatient	182.5	26.6%
	Outpatient	39.7	5.8%
	Pharmacotherapy	17.7	2.6%
	Other medical and non-medical services	67.6	9.9%
	Nursing care	67.0	9.8%
	Special aids and equipment (e.g. crutches, wheel-chairs etc.)	4.4	0.6%
Indirect costs	Costs of absenteeism	40.1	5.8%
	- foregone wages	−13.3	−1.9%
	- loss of added value (e.g. profit, social insurance contributions etc.)	−26.7	−3.9%
	Early retirement	19.3	2.8%
	Family care	207.3	30.2%
TOTAL		685.6	100%

### Number of fractures

In 2008, an estimated total of 119,911 patients with incident osteoporosis-related fractures were treated in Austria (Table 2). 14,871 patients had sustained a hip fracture (12%), 23,934 (20%) a forearm fracture, 9,573 (8%) a humeral fracture, 39,028 (33%) a clinical vertebral fracture, and 32,505 (27%) a rib fracture. For lack of relevant data, morphometric vertebral fractures were not included in our study. Overall, 91,067 cases (76%) were first treated in an outpatient setting, and 28,844 cases (24%) in an inpatient setting.

**Table 2:** Number of fractures, number and costs of bed days, visits, medical and non-medical services

	**Hip**	**Forearm**	**Humerus**	**Clinical vertebral**	**Rib**	**Total**					
	**m**	**f**	**m**	**f**	**m**		**f**	**f**	**m**	**f**	**m**
Fractures											
Cases primarily treated in an inpatient setting	3,415	11,456	878	3,111	770	2,711	1,193	2,355	1,590	1,365	28,844
Cases primarily treated in an outpatient setting	0	0	4,390	15,555	1,348	4,744	11,930	23,550	15,900	13,650	91,067
Cases treated	3,415	11,456	5,268	18,666	2,118	7,455	13,123	25,905	17,490	15,015	119,911
Bed days											
Mean bed days per case	16.6	14.8	3.7	4.9	5.2^1^	7^1^	5.2^1^	7^1^	5.2^1^	7^1^	7,7
Total bed days	56,689	169,549	3,249	15,244	4,004	18,977	6,202	16,482	8,268	9,555	308,219
Costs total (€)^2^	33,559,888	100,373,008	1,923,408	9,024,448	2,370,368	11,234,384	3,671,584	9,757,344	4,894,659	5,656,560	182,465,648
Visits											
Mean number of visits to private practice offices	1.67	2.57	0.33	0.85	0.53	1.82	2.20	2.87	2.20	2.87	1.79
Total number of visits to private practice offices	5,703	29,442	1,738	15,866	1,123	13,568	28,871	74,347	38,478	43,093	252,229
Costs of visits to private practice offices (€)^3^	416,889.3	2,152,210.2	127,047.8	1,159,804.6	82,091.3	991,820.8	2,110,470.1	5,434,765.7	2,812,741.8	3,150,098.3	18,437,939.9
Mean number of visits to hospital outpatient clinics	1.59	1.27	5.83	5.15	1.47	1.71	2.40	1.71	2.40	1.71	2,52
Total number of visits to hospital outpatient clinics	5,430	14,549	30,712	96,130	3,113	12,748	31,495	44,298	41,976	25,676	306,127
Costs of visits to hospital outpatient clinics (€)^4^	290,776.5	779,098.95	1,644,627.6	5,147,761.5	166,701.15	682,655.4	1,686,557.25	2,372,157.9	2,247,814.8	1,374,949.8	16,393,100.85
Other medical and non-medical services											
Physical therapy (mean number of sessions per case)	15.88	14.03	4.17	13.09	25.13	19.98	8.45	8.22	8.45	8.22	12.56
Physical therapy (total number of sessions)	54,230	160,728	21,968	244,338	53,225	148,951	110,889	212,939	147,791	123,423	1,278,482
Costs of physical therapy (€)^5^	1,626,900	4,821,840	659,040	7,330,140	1,596,750	4,468,530	3,326,670	6,388,170	4,433,730	3,702,690	38,354,460
X-rays b (mean number per case)	2.18	2.,17	3.5	4.2	1.33	2.08	2.5	1.96	2.5	1.96	2.43
X-rays (total number)	7,445	24,860	18,438	78,397	2,817	15,506	32,808	50,774	43,725	29,429	304,199
Costs of X-rays (€)^6^	598,578	1,998,744	1,482,415	6,303,118	226,486.8	1,246,682.4	2,637,763	4,082,229	3,515,490	2,366,091	24,457,599
BMD Measurement^7^	1,059	3,551	1,633	5,787	657	2,311	4,068	8,030	5,422	4,655	37,173
Cost of BMD measurement (€)^8^	37,065	124,285	57,155	202,545	22,995	80,885	142,380	281,050	189,770	162,925	1,301,055
Transportation (mean number of units per case)	0.73	0.78	---	0.89	---	0.1	0.55	0.37	0.55	0.37	0.43
Transportation (total number of units)	2,493	8,936	---	16,613	---	746	7,218	9,585	9,620	5,556	60,767
Cost of transportation (€)^9^	142,101	509,352	---	946,941	---	42,522	411,426	546,345	548,340	316,692	3,463,719

^1^ mean values for ICD-10 codes S10-S51, S53-S71, S73-S81.

^2^ Cost per bed day = € 592.

^3^ Cost per visit to private practice office (specialist) = € 73.10.

^4^ Cost per visit to hospital outpatient clinics (speacialist) = € 53.55.

^5^ Cost per session of physical therapy = € 30.

^6^ Cost per unit (i.e. X-ray) = € 80.40.

^7^ In average, 31% of Austrian patients received BMD measurment within first year after fracture (ICUROS-Austria).

^8^ Cost per BMD measurement (spine and hip).

^9^ Average cost per unit (i.e. per ride, as invoiced by a typical Ambulance service) = € 57.00.

### Bed days

Mean bed days per fracture type was 15.7 for hip fracture, 4.3 for forearm fracture, and 6.1 for humeral, clinical vertebral, and rib fractures (Table 2). The estimated total number of bed days for all fractures was 308,219. Inpatient costs were € 182.4 million, accounting for about 45% of direct costs, and 27% of total costs, respectively.

### Number of visits

The total number of visits (not including the primary visit following fracture) to private practice offices and hospital outpatient clinics for all fractures was 252,229 and 306,127, respectively (Table 2). Mean number of visits to private practice offices and hospital outpatient clinics for all fractures was 1.7 and 2.5, respectively. Total costs of visits to private practice offices were € 18,375,371.6, and total costs of visits to hospital outpatient clinics were € 16,393,100.85, accounting for 5.8% of overall cost.

### Other medical and non-medical services

214,958 physical therapy sessions have been utilized following hip fracture, 266,306 following forearm fracture, 202,176 following humeral fracture, 323,828 following clinical vertebral fracture, and 271,214 following rib fracture (Table 2). In total, 1,278,482 physical therapy sessions have been utilized throughout 2008 as a direct consequence of osteoporotic fractures. Total costs of all physical therapy sessions due to osteoporotic fractures were € 38.4 million, representing 5,6% of overall cost.

An estimated 32,305 x-rays have been taken following hip fracture, 96.835 x-rays following forearm fracture, 18,323 x-rays following humeral fracture, 83,582 x-rays following clinical vertebral fracture, and 73,154 x-rays following rib fracture (Table 2). The estimated total number of x-rays as a consequence of osteoporotic fractures was 304,199. Total costs of all x-rays taken as a consequence of osteoporotic fractures were € 24.5 million, accounting for 3.6% of overall cost. It should be noted though that for lack of data, follow-up MRT’s or CT’s have not been included in our study, making it very likely that true costs have rather been higher than shown in this study.

The number of transportation services required as a consequence of fracture was 11,429 for hip fracture, 16,613 for forearm fracture, 746 for humeral fracture, 16,803 for clinical vertebral fracture, and 15,176 for rib fracture (Table 2). In total, 60,766 units of transportation services have been utilized. Total costs of transportation services utilized as a consequence of osteoporotic fractures were € 3.5 million, accounting for 0.5% of overall cost.

### Pharmacotherapy

A total of 22.4% of patients received adequate osteoporosis-specific treatment, which is a very low share from a medical point of view. Patients were treated with either alendronate (12%), risedronate (3.6%), ibandronate (3.6%), zoledronate (1.4%), parathyroid hormone (0.6%; including both teriparatide and 1–84 PTH), Raloxifene (0.4%), strontium ranelate (0.4%), or calcitonin nasal-spray (0.4%) (Table 3). Proportional cost of analgesic treatment was estimated at € 3,354,145, and costs of calcium and vitamin D treatment (without patients treated with alendronate or risedronate) at € 2,185,350. Overall, estimated cost of pharmacotherapy was € 17,920,047, accounting for 2.7% of total costs.

**Table 3:** Costs of pharmacotherapy

**Drug**	**Brand Name(Austria)**	**% patients with osteoporotic fracture (n = 119.911)**	**Total number of patients treated**	**Annual cost of treatment per capita**^**3**^	**Total costs**
Alendronate	Fosamax 10 mg®	2.4% ^1^	2,878	321.0	923,838
	Fosamax 70 mg®	6.7%^1^	8,034	213.0	1,711,242
	Generic Alendronate 70 mg (e.g. Alendronstad®)	2.9% ^1^	3,477	205.2	713,480
		{Total:12.0%}			
Ibandronate	Bonviva 3 mg®	3.6%^2^	4,316	456.4	1,969,822
Risedronate	Actonel 5 mg®	0.5%^2^	600	438.6	263,160
	Actonel 35 mg®	3.1%^2^	3,717	490.2	1,821,330
		{Total: 3.6%}			
Zoledronate	Aclasta 5 mg®	1.4%	1,679	303.1	508,905
Raloxifene	Evista 60 mg®	0.4%	480	438.6	210,528
Strontium Ranelate	Protelos 2 g®	0.4%	480	570.6	273,888
Parathyroid-hormone	Forsteo®/Preotact®	0.6%	720	4,867.6^4^	3,504,672
Calcitonin (nasal-spray)	Calcitonin Novartis Nasal Spray®	0.4%	480	639.0	306,720
Total		22.4%	26,861	---	12,207,585
Analgesics	NSAR (e.g. Diclofenac 50 mg®)	37%	44,367	75.6	3,354,145
Calcium (1000 mg) *+ Vit D (800 IU)*	e.g. Cal-d-Vita® (500 mg/800 IU)	24.3%^5^	29,138	75.0	2,185,350^5^
					5,539,495
				Total	17,747.080

^1^ Estimated proportions of Fosamax daily, once-weekly, and generic forms according to data provided by IMS.

^2^ Estimated proportions of Actonel daily vs once-weekly forms according to data provided by IMS.

^3^ Annual cost of treatment according to version 10 (Oct.) of the Austrian EKO (Erstattungskodex) 2008.

^4^ Forsteo® + Preotact® (mean value applied).

^5^ Without patients who are receiving alendronate or risedronate (calcium + vitamin D tablets already included in price).

### Nursing care and informal care

Average number of nursing care hours per case was 83 hours for hip fracture, 14 hours for forearm fracture, 25 hours for humeral fracture, and 38 hours for clinical vertebral fracture Table 4. The total number of nursing care hours as a consequence of osteoporotic fractures was 3,232,453. Total costs of nursing care hours were estimated at € 97.0 million, thus accounting for 9.8% of overall cost. The average number of family care hours per case was 165 hours for hip fracture, 32 hours for forearm fracture, 84 hours for humeral fracture, and 81 hours for clinical vertebral fracture. The total number of family care hours was 7,536,660. Estimated total costs of (informal) family care hours were € 207.3 millions, accounting for 30.2% of overall cost.

**Table 4:** Costs of nursing care, informal care, and special aids/equipment

	**Hip**	**Forearm**	**Humerus**	**Clinical vertebral**	**Rib**^**1**^	**Costs €**					
	**m**	**f**	**m**	**f**	**m**	**f**	**m**	**f**	**m**	**f**	
Nursing care											
Mean number of nursing care hours per case	108	58	0	14	0	25	17	59	**--------------------**	**--------------------**	**--------------------**
Total number of nursing care hours	368,820	664,448	0	261,324	0	186,375	223,091	1,528,395	**--------------------**	**--------------------**	3,232,453
Total costs (€)^2^	11,064,600	19,933,440	0	7,839,720	0	5,591,250	6,692,730	45,851,850	**--------------------**	**--------------------**	96,973,590
Informal (family care)											
Mean number of family care hours per case	199	130	0	32	5	163	51	111	**--------------------**	**--------------------**	**--------------------**
Total number of family care hours according to fracture type	679,585	1,489,280	0	597,312	10,590	1,215,165	669,273	2,875,455	**--------------------**	**--------------------**	7,536,660
Total costs (€)^3^	18,688,588	40,955,200	0	16,426,080	291,225	33,417,038	18,405,008	79,075,013	**--------------------**	**--------------------**	207,258,151
Special aids and equipment (crutches, wheel-chair etc.)											
Mean expenses (€) per case	49,00	111,67	0,00	5,00	0,00	79,59	31,75	69,88	**--------------------**	**--------------------**	34,7
Total expenses (€)	167,335	1,279,292	0	93,330	0	593,343	416,655	1,810,241	**--------------------**	**--------------------**	4,360,196

^1^ No data available from the ICUROS.

^2^ Estimated cost per hour of nursing care = € 30.

^3^ Estimated cost per hour of family care = € 27.50.

### Absenteeism

Numbers and costs of absenteeism are only related to patients who have not yet retired. In 2008, 5,816 persons have been on sick leave due to osteoporotic fractures, with a mean duration of 16.3 days in men, and 18.9 days in women. In total, 100,841 sick leave days due to osteoporotic fractures have been taken (Table 5). Multiplied by the average daily income of € 128.11 this results to total costs of € 3.3 million €. The 100,841 sick leave days induced a total loss of added value of € 40.1 million, according our calculations based on the official Austrian Input–output table.

**Table 5:** Costs of absenteeism

	**Men**	**Women**	**All**
Persons on sick leave due to osteoporotic fractures	3,490	2,326	5,816
Mean duration (days)	16.3	18.9	17.6
Total days	56,887	43,961	100,848
Mean gross annual income (€)	35,325	21,041	28,183
Daily income (€ year/220 working days)	160.57	95.65	128.11
Costs of absenteeism (€)	9,134,346	4,204,869.6	13,339,215

## Discussion

This study provides for the first time realistic estimates on the burden of different osteoporosis-related fractures in Austria indicating a substantial financial impact for the Austrian society in the year 2008 with a total burden of more than €685 million. With regard to direct costs, 26.6% of all costs (approx. €183 million) were spent on hospitalisation, followed by ambulatory care (13%, i.e. €93.4 million), and pharmacotherapy (12%, i.e. approx. €85 million). 34% of the total costs comprise indirect costs with estimates of informal care being the biggest part (26%, i.e. approx. €186 million). Osteoporosis is also associated with costs due to lost economic value-added which amounts to approx. €40 million.

Although costs of osteoporotic fractures have also been estimated in other cost-of-illness studies, these estimates cannot be directly compared because of differences in resource use and patterns of care as well as different price levels of the treatment of fractures. However, these studies confirm that with respect to direct costs, the most costly fractures are hip fractures followed by vertebral fractures [26-28].

Our study, which also took into account nursing and informal care costs derived from the ICUROS data on utilisation of these resources, shows that vertebral fractures are associated with the highest proportion of these cost factors, which is in line with the results from the KOFOR study [27].

Most published studies on costs of osteoporosis used only hospitalization rates and applied internationally accepted attribution factors [7,10,28-32], but this approach underestimates the total burden of this disease. To minimize deficiencies inherent in under-diagnosis we not only used information drawn from the national hospital discharge register and Statistics Austria but also international data of estimations on the outpatient utilisation of resources due to osteoporotic fractures. One of the strengths of the study presented here lies in the fact that a “real world” estimation regarding the proportion of patients who would be treated after having experienced an osteoporotic fracture in Austria was applied. These estimations have been based on the findings from the ICUROS, in which it was clearly demonstrated that in Austria, irrespective of innumerable awareness programmes that have been performed in the past, only about 22% of those who experience an osteoporotic fracture would receive an adequate pharmacological treatment.

There are, however, several methodological issues to be considered in interpreting the results of our study. Although it is mandatory for all hospitals in Austria to record discharge diagnoses by using the code classes of the International Classification of Diseases (ICD-10), it is possible that patients have multiple registrations for the same diagnosis. Based on this, Dimai et al. used a correction factor of 0.9 to adjust the incidence calculations for hip fractures in Austria [13]. Since there is no information available on age group specific correction factors for other types of fracture than hip, no correction factor was used for these types of fracture.

Another limitation of our study may be the method used to estimate those fractures, which are only partially treated in hospitals. For instance, it has been estimated that only one in three of those who experience a vertebral fracture will come to medical attention [33]. From these, as shown in a Swedish study, only approximately 10% would be hospitalized [16]. Furthermore, treatment of distal forearm fractures is highly variable in different centres in Austria. While some prefer surgical treatment others prefer casting, which is done on an outpatient basis. Likewise, high geographical variations have been noted in international studies on the prevalence of different osteoporotic fractures. With this in mind, our multiplication factors, which were taken according to results from several other national studies, may actually either over- or underestimate the total rates of the different fractures due to osteoporosis in Austria.

Unfortunately, our study has not been designed to include costs for rehab. However, we believe that this limitation of our study would not impose a significant bias, since e.g. in Germany only 4% of total expenses are incurred by rehabilitation costs [12].

In contrast to the high expenditures, significant progress in the treatment and prevention of osteoporosis has been achieved in the past two decades with the development of different compounds with a proven effect on bone mineral density and reduction of fracture risk [34-36].

Despite of this, approximately 50% of patients do not follow their prescribed treatment regimen and/or discontinue treatment within one year [37]. Poor compliance is not only associated with higher fracture rates but also increased morbidity, mortality, and costs [38,39]. Likewise, a German study has shown that only 22% of osteoporotic patients have received treatment; and moreover, 58% (daily dosing) and 43% (weekly dosing) of these patients terminate their treatment [12]. Even though these medications are only indicated in patients with a fracture risk of more than 30%, these numbers reflect a high degree of unawareness and under-treatment of osteoporosis. This has also been shown in a recent Austrian study in nursing homes and senior’s residences, where more than 20% of the patients had a diagnosis of osteoporosis, but only 7.2% of them were prescribed specific treatment [40].

## Conclusions

The results of our cost-of-illness study show that osteoporosis is a costly disease with a significant financial burden to the Austrian society. To improve the awareness of osteoporosis being not only an economic problem, but also a determinant of quality of life in patients affected, appropriate public health strategies need to be applied. In this regard, the substantial socio-economic burden resulting from osteoporotic fractures underlines the importance of pharmacological treatment in those who are at risk of osteoporosis, to prevent first and subsequent fractures.

## Competing interest

The research project was financially supported by Amgen Austria.

## Authors’ contributions

HD carried out the epidemiological studies, and drafted the manuscript. KR participated in the epidemiological studies. MP participated in the coordination of the study, and contributed to the draft. FB performed statistical analyses and participated in the design of the study. US participated in the design and coordination of the study. RM participated in the coordination of the study, participated in its design and helped to draft the manuscript. All authors read and approved the final manuscript.
